# Pain and Pessimism: Dairy Calves Exhibit Negative Judgement Bias following Hot-Iron Disbudding

**DOI:** 10.1371/journal.pone.0080556

**Published:** 2013-12-04

**Authors:** Heather W. Neave, Rolnei R. Daros, João H. C. Costa, Marina A. G. von Keyserlingk, Daniel M. Weary

**Affiliations:** 1 Animal Welfare Program, Faculty of Land and Food Systems, University of British Columbia, Vancouver, British Columbia, Canada; University of Rennes 1, France

## Abstract

Pain is defined as an unpleasant sensory and emotional experience associated with actual or potential tissue damage, but emotional states are difficult to directly assess in animals. Researchers have assessed pain using behavioural and physiological measures, but these approaches are limited to understanding the arousal rather than valence of the emotional experience. Cognitive bias tasks show that depressed humans judge ambiguous events negatively and this technique has been applied to assess emotional states in animals. However, limited research has examined how pain states affect cognitive processes in animals. Here we present the first evidence of cognitive bias in response to pain in any non-human species. In two experiments, dairy calves (n = 17) were trained to respond differentially to red and white video screens and then tested with unreinforced ambiguous colours in two or three test sessions before and two sessions after the routine practice of hot-iron disbudding. After disbudding calves were more likely to judge ambiguous colours as negative. This ‘pessimistic’ bias indicates that post-operative pain following hot-iron disbudding results in a negative change in emotional state.

## Introduction

In the recent literature, emotion has been considered to include behavioural, physiological, cognitive, and subjective components [1]. The first two components are relatively simple to measure in animals (e.g. vocalizations, cortisol levels), and give an indication of emotional intensity but not valence (i.e. pleasant or unpleasantness). This is problematic, as emotional valence is more informing of an animal's welfare status compared to emotional arousal. Because animals are unable to verbally convey their subjective experiences, researchers have turned to the cognitive component of emotion as a method of assessing emotional valence. Emotional responses in humans are associated with changes in cognitive functioning, including attention, memory, and judgement bias [1]. The effect of emotion on judgement making has been explored in a number of areas, including risk-taking, future expectations, and interpretation of ambiguous stimuli. Depressed or anxious people interpret ambiguous stimuli more negatively [2], while people in positive states have more optimistic interpretations [3], [4].

Recent research has attempted to use changes in cognitive processes as a method of assessing emotions of animals [5]. The first published study on cognitive bias in animals [6] focused on judgements of ambiguous stimuli presented in an operant discrimination task. Rats were housed in predictable and unpredictable housing conditions; the latter condition was intended to induce a negative affective state in the rats. Compared to the control rats, rats in the unpredictable housing condition showed fewer positive responses to the ambiguous stimulus. More recent studies have examined different species exposed to various other affect manipulations [5]–[12] and have found similar biases, illustrating that judgement biases can be useful in identifying emotional states in animals.

Pain is defined by the International Association for the Study of Pain [13] as “an unpleasant sensory and emotional experience associated with actual or potential tissue damage.” Indeed, pain is one of the most highly studied emotions in animals [14], and the rich interplay between pain and cognitive processes is well studied in humans [15], [16].

Research on pain assessment in animals has focused on behavioural and physiological measures, but these measures only provide an understanding of the arousal of the emotional experience. Studies in rodents have also shown cognitive changes in response to pain, including attentional biases [17], conditioned place aversion learning [18] and social modulation [19]. Research on negative judgement bias resulting from pain has been limited to the human literature [20]; we hypothesize that animals in pain exhibit similar judgement biases. Such biases may be functional in that animals experiencing pain are likely more vulnerable to fitness threats and thus may preferentially benefit from interpreting ambiguous stimuli as threatening [21].

Removal of horn buds from dairy calves (i.e. disbudding, also termed dehorning), like other routine surgeries on commercial farms, is commonly performed without use of post-operative analgesics [22]. Post-operative pain from disbudding is associated with behavioural (e.g. head rubbing, head shaking, ear flicking, vocalizations) and physiological changes (e.g. plasma cortisol concentrations) that persist for at least as long as 24 h after the procedure [22]–[24]. The aim of this study was to assess whether the pain-induced changes in emotional state in calves are associated with a judgement bias. We predicted that calves experiencing pain after disbudding would exhibit a pessimistic bias in their responses to ambiguous stimuli.

## Materials and Methods

### (a) Ethics Statement

This study was approved by the Canadian Council on Animal Care (Protocol number: A12-0337). All disbudding surgeries were performed with a sedative and local anaesthetic to minimize the pain calves experienced during disbudding.

### (b) Experiment 1 and 2

Our study included two experiments. The aim of the two experiments was identical, but the there were a number of minor methodological changes in Experiment 2 designed to improve the efficiency of training.

### (c) Animals and experimental testing pen

#### Experiment 1

**Figure 1:**
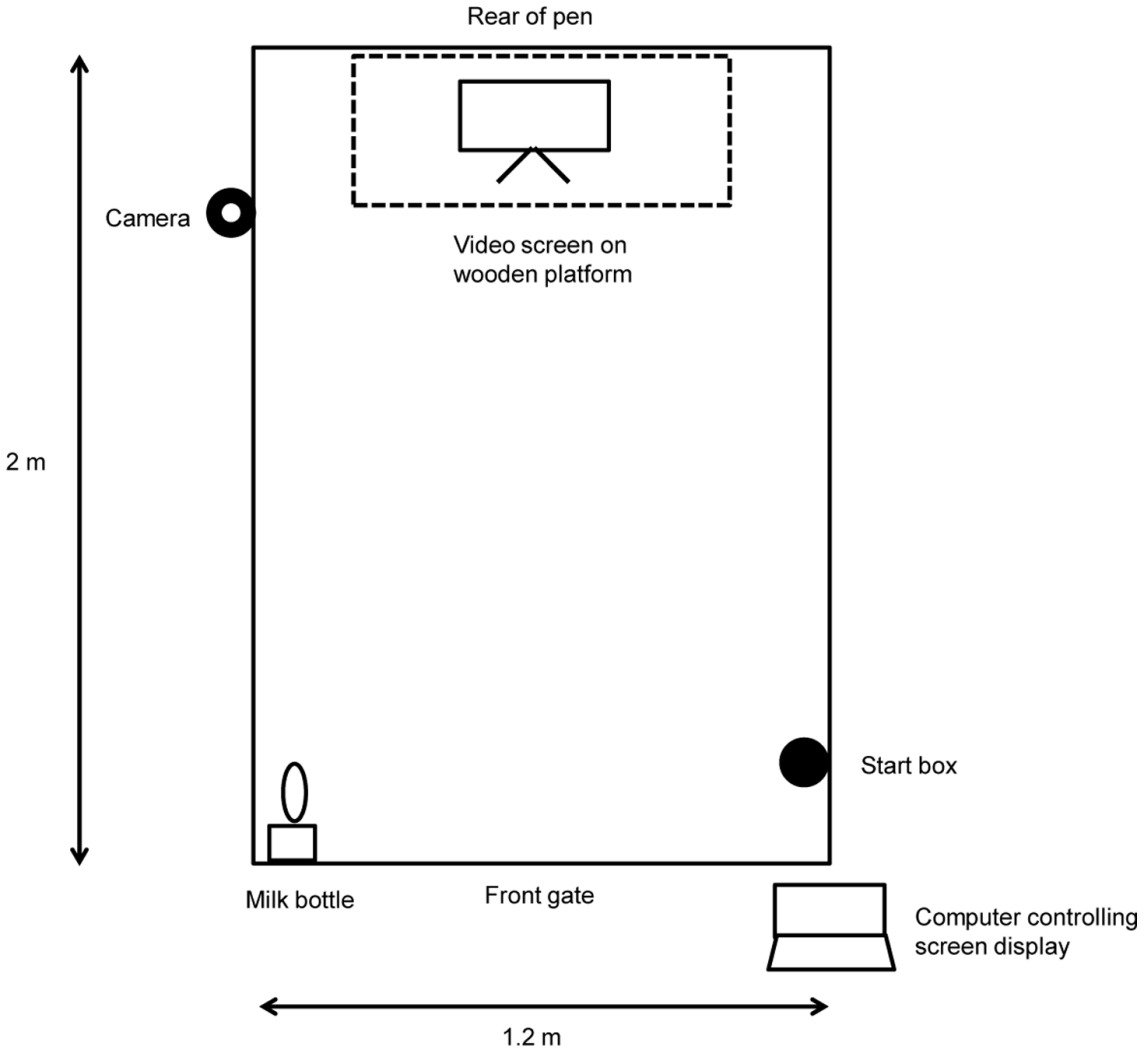
Experimental apparatus for training and testing in the judgement bias task. Calves entered through the front gate to begin each session. The computer controlled the display of positive, ambiguous and negative screens.

We used eight male Holstein calves starting at approximately three days of age. Calves were housed in individual sawdust bedded pens (1.2 m×2.0 m). Management of the calves from birth to enrolment followed standard farm protocol. A separate pen, identical to those the calves were housed in, was used for the cognitive bias task (Fig. 1). A 48-cm computer monitor was fastened at the rear of the pen, approximately 50 cm above the pen floor. Images on the monitor were controlled using a laptop computer. All calves were handled, trained and disbudded by the same person, and calves were trained and tested in the cognitive bias task at the same time each day, twice per day. Training and testing sessions lasted approximately 15 min per calf. Although there was individual variability in learning speed, each step of the training process was tailored to the individual calf (i.e. calves did not proceed to the next stage until the specified criterion was met).

#### Experiment 2

Nine male Holstein calves were enrolled at approximately five days of age. A 38-cm video monitor was used to present training and test screens. All calves were handled, trained and disbudded by two previously trained individuals.

### (d) Initial training

#### Experiment 1

Calves were trained in a go/no-go task to discriminate between two colours displayed on a video screen using the backwards-chaining method [25]. The animals were alternatively assigned either red or white as the positive training stimulus. Calves were clicker-trained to “nose-touch” the video screen (i.e. press nose to video screen) when the positive colour was displayed. Calves were initially conditioned to the sound of the click signalling the arrival of milk (available for 5 s with calves consuming on average 0.14 L), and after ten trials of clicker conditioning, calves were required to nose-touch the video screen by walking increasing distances to the screen before hearing the click and receiving milk. Five consecutive unassisted trials were required before increasing the walking distance to the screen until the calf was able to walk the full distance (1.75 m) from the milk bottle to the video screen and back to receive milk. Training to the positive screen continued until the calves correctly nose-touched the screen without assistance over 2 consecutive sessions. The negative colour was then introduced at a rate of 35% within training sessions; calves were given a 1 min “time-out” (no opportunity to drink milk) if they nose-touched this screen. During this stage of training, positive and negative screens were displayed until the calf showed the correct response (i.e. nose touch the screen in the case of the positive colour and avoid touching the screen for at least 3 s in the case of the negative colour). A training session ended when the calf had consumed 4 L of milk (half the total daily allowance), so the number of screens or “trials” presented in any given session for each calf varied slightly (28.6±7.7, mean ± SE).

Once the learning criterion was reached (80% correct responses over 3 consecutive sessions), calves were clicker trained in the same manner to nose-touch a start box; touching the start box turned on the video screen that the calves then needed to nose-touch. For training purposes, the location of the start box was initially mounted very close to the video screen (30 cm), then at an intermediate distance from the video screen (80 cm), then finally at the opposite end of the pen relative to the video screen (145 cm).

#### Experiment 2

All calves were assigned to the white screen colour as the positive training stimulus, and the red screen colour as the negative training stimulus. Calves received 20 trials of clicker conditioning, and were then assisted to nose-touch the positive screen and subsequently return to the rear of the pen to receive the milk reward from the milk holder. This eliminated the intermediate training steps performed in Experiment 1. Training to the positive screen continued until the calves correctly nose-touched the screen without assistance in 85% of positive trials (i.e. at least 17 out of 20 positive screens) over two consecutive sessions. The negative screen colour was then introduced at a rate of 9, 17 and 24% (2, 4 and 6 negative screens displayed randomly among 20 positive screens) over three consecutive sessions. The number of negative screens within a session was capped at 6 (24%) to prevent frustration. Calves received a noise cue and 1-min “time-out” (no opportunity to drink milk) in response to nose-touches for the negative screen. Training to the negative screen continued until the calves avoided all negative screens over two training sessions. The start box was then introduced, starting at the farthest distance (145 cm) from the video screen.

### (e) Discrimination training

#### Experiment 1

Once calves were trained to nose-touch the video screen after nose-touching the start box (correctly perform the sequence without assistance over 2 consecutive sessions), positive and negative screens were displayed for 3 s from the time the calf nose-touched the start box. Calves were returned to the start box following a time-out for nose-touching the negative screen. The percentage of trials where the negative screen was presented was gradually increased to 50% and the reinforcement rate for the positive screen was gradually reduced to 50%. Punishment rate for negative screen responses continued at 100%. The use of partial reinforcement is similar to approaches used in other cognitive bias studies (e.g. [8]). The reason for reducing positive reinforcement was to reduce the likelihood of extinction of responses to the ambiguous screens. Punishment was not reduced as calves rarely approached the negative screens at this stage of their training. Calves completed on average 30.5±5.5 (mean ± SE) trials per session, consuming 4 L of milk per session, and required an average of 14.5±5.0 (mean ± SE) training sessions in order to reach this discrimination criterion. For a visual of calves performing the discrimination task, see Video S1.

#### Experiment 2

Positive and negative screens were displayed for 4 s from the time the calf nose-touched the start box following successful start box training (correctly performing the sequence without assistance over 2 consecutive sessions). The percentage of negative screens presented was gradually increased to 50% (8, 12, 16 and 20 negative screens among 20 positive screens over 4 consecutive sessions), and the reinforcement rate for the positive screen was gradually reduced to 50% (from 20 to 15 to 10 reinforced screens out of 20 total positive screens) across two training sessions.

### (f) Judgement bias testing

#### Experiment 1

Testing began once calves reached the discrimination criterion (90% correct over 3 consecutive sessions, using 50% positive screens and a 50% reinforcement rate). Ambiguous screen colours were introduced randomly at a rate of 24%, with each ambiguous colour appearing in 8% of trials. The three ambiguous screens were 25% red, 50% red, and 75% red as generated using Adobe Photoshop Elements [26] by adjusting the saturation level of 100% red. Responses to ambiguous probe screens were neither rewarded nor punished. All screen colours were pseudo-randomly displayed in the sequence (no more than two of the same colour screen in succession and no more than 4 unreinforced screens in succession). Calves were tested in 3 sessions before disbudding (26 h, 16 h, and 2 h before disbudding) and 2 sessions after disbudding (6 h and 22 h). A session ended when the calf drank 4 L of milk or when all 60 screens of the sequence had been presented (whichever occurred first), resulting in an average of 55 trials per session. The calf's response (nose-touch the screen or not) was recorded; a “nose-touch” was classified as coming to within 10 cm of the video screen as measured using video. Calves were never punished during testing; this was to reduce the risk that responses to the probes immediately after the negative training screen would be affected by the experience of punishment.

#### Experiment 2

Testing began once calves reached the discrimination criterion (85% correct over 3 consecutive sessions, using 50% positive screens and a 50% reinforcement rate). Calves were tested in 2 sessions (16 h and 2 h) before disbudding and 2 sessions after disbudding (6 h and 22 h). A session ended when all 60 screens of the sequence had been presented.

### (g) Disbudding procedure

Calves were disbudded following the standard operating procedure for our farm. Calves were sedated with an intramuscular injection of xylazine (Rompun, 2%, Bayer Inc., Ontario; 0.25 mg/kg body weight; half life 30 min) 2 h after the last testing session before disbudding. A local anaesthetic (4 mL per side of 2% Lidocaine; Ayerst Veterinary Labs, Ontario; half life 90 min) was applied subcutaneously to the cornual nerve of each horn bud (located under and along the occipital groove), and 5 min later an electric hot-iron (Rhinehart X-30) was applied to each horn bud for approximately 15 s. Calves were allowed 6 h to recover from sedation before testing resumed.

### (h) Statistical analysis

The percentage of screens approached for each of the training and ambiguous screens was calculated for each calf and each session. Residuals were examined to verify normality and homogeneity of variances. The multiple sessions before and after disbudding did not differ for any of the training or ambiguous screens, so we pooled sessions within each phase relative to disbudding (data deposited in Dryad repository [27]).

To determine if calves continued to respond as trained to positive and negative training screens following disbudding, we tested the effects of disbudding, experiment and screen using a mixed model that specified calf as a random effect and used an autoregressive covariance structure. A second identical model was used to compare responses to the probe screens before and after disbudding. All 2-way interactions were tested but these were never significant and are not reported below. All analysis was performed with SAS software [28].

## Results

### (a) Discrimination

The calves learned to discriminate between the positive and negative screens: the percentage of positive and negative screens approached before disbudding averaged 98±1% and 3±1% (mean ± standard error), respectively. Discrimination performance remained high after disbudding (98±1% and 2±1% for positive and negative screens), with no effect of disbudding on response (*p* = 0.4).

### (b) Response to probes

**Figure 2:**
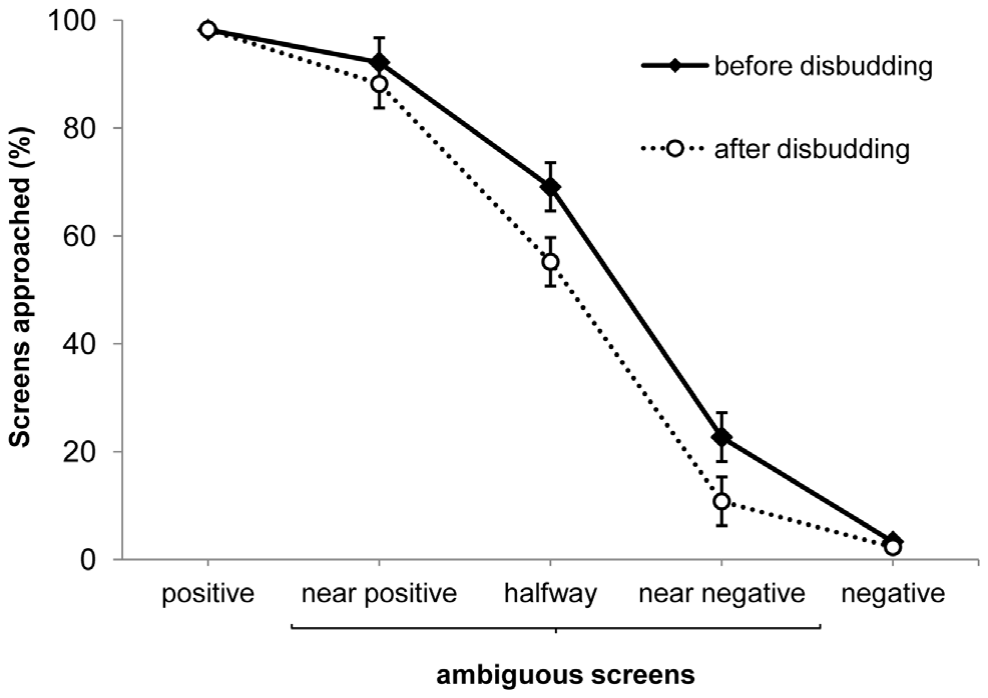
Mean ± SE approach responses of calves to each screen colour before and after disbudding. Calves were trained to approach the positive screen colour for a milk reward, and to avoid approaching the negative screen colour, and were tested with unreinforced ambiguous colours. Calves responded to the ambiguous colours less frequently after versus before disbudding (*p* = 0.0004); as expected, there was no effect of disbudding on the responses to positive and negative training colours.

When tested with ambiguous intermediate screen colours before disbudding, the calves treated the near-positive and near-negative screen colours as being similar to the training screens, approaching these screens in 92% and 23% of trials, respectively; calves were more ambivalent about the halfway red screen, approaching it in 69% of the trials (Fig. 2). After disbudding calves approached the ambiguous screens less frequently compared to before disbudding (*p* = 0.0004). Numerically, this negative bias was weakest for the near-positive probe (with calves approaching this probe 4% less frequently after disbudding) and more prominent for the halfway and near-negative probes (declining 14 and 11% respectively).

Individual calves differed somewhat in their responses to the probes (Table 1), but most calves (13 of 17) responded less frequently to the probe screens after disbudding compared to before. Three of the 17 calves showed no difference and only 1 calf responded more frequently to the probes after disbudding.

**Table 1:** Calf approach responses to each screen before and after disbudding (%).

		Screen colour				
Phase of testing	Calf	Positive	Near positive	Halfway	Near negative	Negative
Before disbudding^a^	1	90	45	13	11	1
	2	98	67	17	25	14
	3	98	92	42	33	8
	4	100	100	83	8	0
	5	97	87	60	0	3
	6	96	93	40	20	5
	7	100	100	80	27	5
	8	97	93	70	11	8
	9	100	100	90	20	0
	10	100	100	50	10	0
	11	96	100	100	60	0
	12	100	100	90	40	2
	13	98	100	90	10	0
	14	98	100	100	10	2
	15	100	100	100	20	0
	16	100	100	80	40	0
	17	100	100	80	50	7
After disbudding^b^	1	91	23	13	0	0
	2	100	88	0	13	12
	3	95	100	50	0	0
	4	100	100	88	0	0
	5	98	100	40	0	5
	6	100	80	0	0	0
	7	100	70	50	10	5
	8	98	80	40	13	0
	9	100	100	70	10	0
	10	100	90	60	0	0
	11	93	100	70	0	0
	12	100	100	90	40	5
	13	100	100	40	10	0
	14	98	80	60	20	0
	15	100	100	100	10	7
	16	98	100	90	30	2
	17	100	100	90	40	5

a Before disbudding: Sessions have been pooled and averaged for each calf (Calf 1–8, Experiment 1: 3 sessions at 26, 16 and 2 h before disbudding; Calf 9–17, Experiment 2: 2 sessions at 16 and 2 h before disbudding).

b After disbudding: Sessions have been pooled and averaged for each calf (all calves: 2 sessions at 6 and 22 h after disbudding).

## Discussion

Calves exhibited a negative judgement bias for at least 22 hours after hot-iron disbudding; calves were less likely to approach ambiguous screen colours in two test sessions after the procedure. This ‘pessimistic’ negative bias is indicative of a negative emotional state in calves following hot-iron disbudding.

These results provide the first evidence of judgement bias associated with pain in non-human animals. Previous studies examining depression and anxiety have shown different types of judgement bias. In humans, depression is associated with a decreased expectation of positive events, while anxiety is associated with an increased expectation of negative events [29], [30]. Studies in animals have reported both types of biases, interpreted based upon their proximity to the “positive” or “negative” reference stimuli in the judgement bias task.

The strongest bias observed in this study was at the halfway and near-negative colours, indicating an increased expectation of negative events. A more pronounced bias at the near-negative probes was also described by Burman et al. [31], Matheson et al. [32] and Pomerantz et al. [6] who induced negative emotional states in rats, starlings, and monkeys, respectively. Based on the direction of the bias, both Burman et al. [31] and Pomerantz et al. [6] proposed that the emotion experienced by the animals was negatively valenced and of high intensity, similar to anxiety. Following this reasoning, we suggest that the pain experienced by these calves was associated with an anxiety-like rather than depression-like emotional state.

The post-operative pain associated with hot-iron disbudding is known to persist at least 24 h (reviewed by [24]). Calves exhibit elevated plasma and salivary cortisol levels and heart rate for up to several hours after hot-iron disbudding [33], and have higher frequencies of head shaking, ear flicking, and head rubbing for up to 24 h after hot-iron disbudding [22], [23]. When given a post-operative analgesic such as ketoprofen or meloxicam following hot-iron disbudding, calves have a marked reduction in ear flicks and head shakes [34], [35] and lower plasma cortisol and heart rates [36] compared to calves without post-operative medication.

In our study, we tested calves' judgement biases at 6 h (the peak of pain behaviours and cortisol levels) and 22 h (toward the end of the post-operative pain period) following disbudding. Interestingly, there was no difference in responses to the ambiguous screens between these two sessions following disbudding, suggesting that the impact of disbudding on the emotional state of calves persists for at least 22 h.

Decreased feeding motivation after disbudding cannot explain the reduced response to the probes as there was no difference in the number of responses to the reinforced training screen after disbudding showing that calves continued to be motivated to drink milk. It is possible that the reduced responding to the probe screens after disbudding was due to calves learning to not respond to the unreinforced probes, but we used a low rate of probes and partial reinforcement specifically to prevent this type of learning and we found no evidence of reduced responding to the probes over multiple test sessions before or after disbudding. However, future studies should include test sessions in the days following disbudding to ensure that calves return to baseline. Finally, the negative bias may have resulted from recovery from the sedative (or the local anaesthetic, or some other part of the procedure) rather than from the pain of disbudding per se. Previous work has shown that calves exhibit little or no behavioural responses during recovery from xylazine and local anaesthetic when disbudding does not occur [23] but no research has investigated how calves *feel* when recovering from these drugs; we encourage further work on this topic. Future studies should include a sham condition where calves are exposed to the xylazine and local block but are not disbudded. We also encourage future research to include a treatment where animals receive post-operative pain control, for example a non-steroidal anti-inflammatory drug such as ketoprofen or meloxicam.

To conclude, this study provides the first evidence of judgement bias in animals following a painful procedure. These results support the use of cognitive bias tasks in the assessment of animal emotions.

## Supporting Information

Video S1 **Calf performing the discrimination task.** This calf was trained to respond to the red “positive” screen for a milk reward; visits to the white “negative” screen were not rewarded. Calves started a trial by touching the start box on the right side of the pen.
(M4V)
