# Correction: Pain and Pessimism: Dairy Calves Exhibit Negative Judgement Bias following Hot-Iron Disbudding

**DOI:** 10.1371/journal.pone.0096135

**Published:** 2014-04-21

**Authors:** The PLOS ONE Staff

There is an error in the entry for Reference 6. The correct Reference 6 is:

“Harding EJ, Paul ES, Mendl M (2004) Animal behaviour - cognitive bias and affective state. Nature 427:312.”

## References

[1] Neave HW, Daros RR, Costa JHC, von Keyserlingk MAG, Weary DM (2013) Pain and Pessimism: Dairy Calves Exhibit Negative Judgement Bias following Hot-Iron Disbudding. PLoS ONE 8(12): e80556 doi:10.1371/journal.pone.0080556 24324609 10.1371/journal.pone.0080556 PMC3851165

